# Malnutrition, Skeletal Muscle Loss and Mucosal Toxicity in Head and Neck Cancer: Nutritional Targets Beyond Energy Replacement

**DOI:** 10.3390/nu18050737

**Published:** 2026-02-25

**Authors:** Réka Fritz, Zoltán Tóbiás, Zsófia Bere, Péter Fritz

**Affiliations:** 1 Department of Oto-Rhino-Laryngology and Head-and Neck Surgery, University of Szeged, 6725 Szeged, Hungaryberezsofia@gmail.com (Z.B.); 2 Faculty of Humanities and Social Sciences, Károli Gáspár University of the Reformed Church in Hungary, 1088 Budapest, Hungary; fritz.peter@kre.hu

**Keywords:** head and neck cancer, malnutrition, skeletal muscle loss, low skeletal muscle mass, mucositis, immunonutrition, enteral nutrition, body composition, inflammation, microbiome

## Abstract

Head and neck cancer represents one of the most nutritionally vulnerable oncologic populations, driven by tumor-related functional impairment, treatment toxicities, and complex metabolic alterations. Malnutrition and skeletal muscle loss are highly prevalent at diagnosis and frequently worsen during therapy, impairing treatment tolerance, functional status, and clinical outcomes. This narrative review synthesizes clinical and mechanistic evidence on the interrelated roles of malnutrition, low skeletal muscle mass, mucosal toxicity, and systemic inflammation across the perioperative, definitive treatment, and post-acute recovery phases. Particular emphasis is placed on the limitations of body mass index-based assessment and the importance of integrating validated screening tools with context-appropriate phenotypic evaluation of muscle depletion. Beyond conventional energy replacement, we examine the evidence supporting perioperative immunonutrition, discuss the contextual limitations of immune-modulating strategies during chemoradiotherapy, and consider emerging adjunctive metabolic approaches. Mucositis is conceptualized not only as a local toxicity but also as a contributor to reduced intake and inflammation-driven catabolism. The post-treatment phase is highlighted as a critical period for continued monitoring of body composition and functional recovery. Collectively, the available evidence supports a shift from weight-centered nutritional paradigms toward an integrated, body composition-oriented and inflammation-aware framework for supportive care in head and neck oncology.

## 1. Introduction

Patients with head and neck cancer (HNC) represent one of the most nutritionally vulnerable populations in oncology. Tumor localization frequently compromises mastication, swallowing, and oral intake even before treatment initiation, while multimodal therapies—including surgery, radiotherapy, and chemoradiotherapy—further aggravate functional impairment through mucosal toxicity, pain, xerostomia, and sensory alterations. Consequently, clinically relevant nutritional decline is often already present at diagnosis and may accelerate during treatment, with direct implications for treatment feasibility and outcomes [1,2].

HNC is biologically and clinically heterogeneous. Tumors of the oral cavity, oropharynx, hypopharynx, and larynx differ in anatomical impact, therapeutic approach, and metabolic burden. Extensive resections with reconstruction induce acute surgical stress and wound-healing demands, whereas definitive chemoradiotherapy is characterized by cumulative mucosal injury and sustained inflammatory activation. These modality-specific contexts shape nutritional trajectories and patterns of body composition change and should be considered when interpreting outcome data.

Nutritional impairment in HNC extends beyond involuntary weight loss. Reduced intake frequently coexists with systemic inflammation and altered protein metabolism. Imaging-defined depletion of skeletal muscle mass has emerged as a clinically relevant prognostic factor associated with treatment-related toxicity, postoperative complications, and inferior survival [3,4]. However, contemporary consensus definitions of sarcopenia require not only reduced muscle mass but also impaired muscle strength or performance. In the HNC literature, muscle mass is often quantified through imaging-based techniques, whereas objective functional assessment is less consistently incorporated. Maintaining terminological precision is therefore essential to avoid conflating isolated muscle depletion with fully defined sarcopenia.

Traditional reliance on body mass index (BMI) alone is increasingly recognized as insufficient in this setting. BMI does not differentiate between fat mass and lean tissue and may remain stable despite progressive muscle depletion. At the same time, validated screening instruments—such as the Patient-Generated Subjective Global Assessment (PG-SGA) and the Mini Nutritional Assessment (MNA)—provide structured approaches to identifying patients at nutritional risk. The Global Leadership Initiative on Malnutrition (GLIM) criteria further refine diagnostic classification by integrating phenotypic and etiologic components, including inflammation and reduced intake. Rather than replacing established tools, GLIM may complement them by enhancing phenotypic characterization when feasible in routine care.

This review synthesizes current clinical and mechanistic evidence on malnutrition, low skeletal muscle mass, and treatment-related toxicity in HNC. Particular emphasis is placed on structured assessment strategies and their implications for perioperative, definitive treatment, and post-acute recovery phases. By aligning pathophysiology with assessment methodology, we aim to provide a coherent framework for supportive nutritional care in head and neck oncology.

## 2. Malnutrition and Low Skeletal Muscle Mass in Head and Neck Cancer

Malnutrition affects a substantial proportion of patients with head and neck cancer, with reported prevalence ranging from 40% to 80%, depending on tumor site, disease stage, and assessment methodology [1,2]. Structural disruption of alimentation combined with treatment-related toxicity and inflammatory activation contributes to this high burden. Compared with many other solid tumors, HNC directly compromises the functional integrity of the upper aerodigestive tract, predisposing patients to rapid nutritional deterioration.

The clinical consequences of malnutrition extend beyond weight loss. Undernourished patients demonstrate reduced tolerance to radiotherapy and chemotherapy, higher rates of postoperative complications and infections, prolonged hospitalization, and diminished quality of life [1,5]. These complications may necessitate dose reductions or treatment interruptions, potentially affecting oncologic efficacy.

Reduced skeletal muscle mass has emerged as an important prognostic marker in HNC. In oncologic populations, cross-sectional muscle area at the third lumbar vertebra (L3) is generally considered the reference anatomical site for estimating whole-body skeletal muscle mass. However, abdominal imaging at L3 is rarely available in routine diagnostic work-up of head and neck cancer. Consequently, cross-sectional muscle area at the C3 vertebral level on routine head and neck CT imaging has been widely adopted as a pragmatic surrogate for skeletal muscle estimation in this population [4,6].

It is important to emphasize that most HNC studies quantify muscle mass alone without systematic evaluation of muscle strength or performance. According to contemporary consensus criteria, sarcopenia requires both reduced muscle mass and impaired muscle function. Much of the existing HNC literature therefore more precisely describes low skeletal muscle mass rather than fully defined sarcopenia. Preserving conceptual clarity in this regard is critical for interpretation of prognostic associations and for comparability across studies.

The entity of sarcopenic obesity—characterized by excess adiposity in the presence of muscle depletion—has been described in oncologic populations with higher baseline BMI [3]. In HNC, its prevalence and prognostic significance remain incompletely characterized, with heterogeneous findings across tumor sites and treatment modalities. Careful contextual interpretation is therefore required.

From a biological perspective, muscle depletion in HNC reflects the combined impact of insufficient intake and inflammation-mediated catabolism. Cytokine activation promotes proteolysis and suppresses anabolic signaling, while reduced dietary protein intake limits compensatory muscle protein synthesis. Diminished physical activity during intensive treatment may further contribute to negative protein balance.

In summary, malnutrition and low skeletal muscle mass represent interrelated yet distinct dimensions of nutritional vulnerability in HNC. Accurate characterization requires terminology and assessment strategies that extend beyond body weight alone.

## 3. Nutritional Screening, Diagnostic Frameworks and Body Composition Assessment

Body mass index remains a widely used and pragmatic indicator of nutritional status in oncology. Its strengths lie in simplicity and universal availability. However, BMI does not distinguish between adipose and lean tissue compartments and may remain within the normal range despite clinically meaningful muscle depletion, particularly in patients with preserved baseline adiposity [3]. Reliance on BMI alone may therefore underestimate nutritional impairment in HNC.

Validated screening instruments are central to routine practice. The Patient-Generated Subjective Global Assessment (PG-SGA) integrates weight history, dietary intake, symptom burden, and functional status and is widely applied in oncology. The Mini Nutritional Assessment (MNA) offers additional value in older or frail populations. BMI-adjusted weight loss grading systems provide complementary prognostic stratification. These tools are feasible in high-throughput clinical environments and demonstrate associations with clinically relevant outcomes.

The Global Leadership Initiative on Malnutrition (GLIM) criteria were introduced to harmonize diagnostic classification by combining phenotypic criteria (weight loss, low BMI, reduced muscle mass) with etiologic criteria (reduced intake and/or inflammation) [7]. In head and neck cancer, where impaired intake and inflammatory activation frequently coexist, this integrated framework is conceptually appropriate. Implementation, however, may require access to objective muscle mass assessment, which is not uniformly available. GLIM should therefore be regarded as a complementary diagnostic approach that refines phenotypic characterization rather than replacing established screening instruments.

Objective body composition assessment enhances diagnostic precision. As noted above, skeletal muscle cross-sectional area at the C3 level on routine head and neck CT imaging provides a practical surrogate for whole-body muscle mass when L3 imaging is unavailable [4]. Bioelectrical impedance analysis and other non-invasive methods may support longitudinal monitoring where accessible. Institutional resources and workflow considerations inevitably influence feasibility of systematic implementation.

Evidence from anatomically related surgical contexts further illustrates the limitations of weight-based monitoring. In patients undergoing tonsillectomy, reductions in fat-free mass have been documented despite minimal change in BMI [8]. Although etiologically distinct from oncologic disease, this observation underscores how localized impairment of the upper aerodigestive tract may influence body composition without overt weight change.

In clinical practice, a stepwise approach is pragmatic. Initial screening using validated tools such as PG-SGA or MNA can identify patients at risk. Where feasible, additional phenotypic assessment—including evaluation of muscle mass—may refine risk stratification and inform individualized intervention.

Taken together, nutritional assessment in HNC should integrate pragmatic screening with context-appropriate phenotypic refinement. No single metric is sufficient in isolation; the objective is timely identification and accurate characterization of nutritional vulnerability to guide targeted supportive care.

## 4. Nutritional Interventions: From Intake Support to Muscle-Oriented Management

Nutritional intervention in head and neck cancer (HNC) has traditionally focused on restoring energy balance and preventing excessive weight loss. Although adequate caloric provision remains essential, growing evidence indicates that energy replacement alone does not reliably prevent progressive loss of skeletal muscle mass in cancer populations [6,9]. In HNC, where reduced intake frequently coexists with systemic inflammation and treatment-related metabolic stress, this limitation becomes particularly relevant.

Within the conceptual framework outlined in Section 3, reduced intake and inflammation represent interacting drivers of malnutrition. Nutritional strategies must therefore address both insufficient substrate delivery and the catabolic milieu that impairs effective utilization of those substrates. The clinical objective extends beyond weight stabilization toward attenuation of lean tissue depletion, which has been associated with adverse oncologic and functional outcomes [4,9].

### 4.1. Timing and Early Intervention

In routine clinical practice, escalation of nutritional support is often reactive, initiated only after clinically apparent weight loss or severe dysphagia has developed. However, observational and interventional data suggest that earlier initiation of structured nutritional care—beginning at diagnosis or at the onset of treatment—may improve treatment adherence and reduce unplanned interruptions [1,5].

Importantly, much of the available literature in HNC documents reduced skeletal muscle mass rather than fully defined sarcopenia, as objective functional measures are not consistently incorporated [4]. Accordingly, prevention of further lean tissue loss represents a realistic and clinically meaningful target during active treatment, even when reversal of established functional impairment may not be achievable.

### 4.2. Oral Nutritional Supplementation: Scope and Limitations

Oral nutritional supplements (ONS) constitute a central component of supportive care during radiotherapy, chemoradiotherapy, and postoperative recovery. When combined with individualized dietary counseling, ONS have been shown to improve total energy and protein intake and mitigate the magnitude of weight loss in cancer patients [1,8].

Nevertheless, preservation of body weight does not necessarily reflect preservation of skeletal muscle. Inflammatory activation and anabolic resistance may limit the efficiency of muscle protein synthesis despite adequate caloric intake [6,9]. Therefore, while ONS play an important role in preventing severe energy deficit, expectations regarding their capacity to fully counteract treatment-associated muscle depletion should remain measured.

### 4.3. Enteral Nutrition and Treatment Continuity

When oral intake becomes insufficient due to dysphagia, pain, or mucosal injury, enteral nutrition via nasogastric tube or percutaneous endoscopic gastrostomy is frequently required. In HNC, the primary clinical aim of enteral feeding is to ensure treatment continuity and prevent critical nutritional deterioration that could necessitate dose modification or interruption [2,5].

Evidence indicates that appropriately timed enteral nutrition reduces severe weight loss and supports overall nutritional status during intensive treatment [2]. However, similar to oral supplementation, enteral feeding does not inherently reverse inflammation-driven catabolism. Even under conditions of adequate caloric delivery, low muscle mass may persist or progress if systemic metabolic stress remains unmitigated [4,9].

### 4.4. Heterogeneity of Head and Neck Cancer and Body Composition Trajectories

Head and neck cancer encompasses heterogeneous tumor sites and treatment pathways. Oral cavity tumors frequently produce early mechanical impairment of mastication and swallowing, whereas non-oral cavity malignancies may exhibit different temporal patterns of intake limitation and toxicity. These distinctions influence the trajectory of nutritional decline and the relative contributions of reduced intake versus treatment-related inflammation.

Body composition phenotypes also require cautious interpretation. Although skeletal muscle depletion is consistently associated with adverse outcomes in HNC [4], the prevalence and prognostic relevance of sarcopenic obesity in this population remain incompletely characterized [3]. Extrapolation from other oncologic entities should therefore be undertaken carefully, with recognition of disease-specific variability.

### 4.5. Conceptual Transition Toward Context-Specific Metabolic Support

Taken together, current evidence supports a stepwise and context-sensitive approach. Pragmatic screening tools and conventional measures such as BMI and weight loss remain valuable in routine care (Section 3), while more detailed phenotypic assessment refines risk stratification. Nutritional interventions should then be tailored according to treatment modality, metabolic burden, and individual patient characteristics.

Energy replacement is necessary but rarely sufficient. The interaction between reduced intake, inflammatory activation, and muscle catabolism underscores the need for strategies that extend beyond caloric adequacy alone [6,9]. This rationale provides the conceptual bridge to adjunctive metabolic approaches discussed in the subsequent section, where the potential role and limitations of immunonutrition are considered in treatment-specific contexts.

## 5. Immunonutrition and Adjunctive Metabolic Support

Immunonutrition refers to enteral formulations enriched with substrates such as arginine, omega-3 fatty acids, nucleotides, and antioxidants, designed to modulate inflammatory and immune responses in the context of surgical or oncologic stress. The rationale for immunonutrition is particularly relevant in head and neck cancer (HNC), where surgical trauma, impaired intake, and systemic inflammatory activation frequently coexist.

For conceptual clarity, it is important to distinguish between immunonutrition and broader forms of adjunctive metabolic support. In this review, the term immunonutrition refers specifically to commercially formulated enteral preparations enriched with immune-modulating substrates—most commonly arginine, omega-3 fatty acids, nucleotides, and antioxidants—primarily evaluated in perioperative settings. These formulations are designed to attenuate acute inflammatory responses and support wound healing during surgical stress.

In contrast, adjunctive metabolic support denotes more targeted strategies aimed at influencing muscle protein turnover, anabolic signaling, or inflammatory modulation through specific substrate provision, such as branched-chain or essential amino acids. Although practical overlap may occur—particularly when enriched formulas contain amino acid components—the clinical rationale, mechanistic emphasis, and evidence base differ. Maintaining this distinction helps avoid conflation of perioperative immunonutrition protocols with emerging muscle-directed metabolic strategies.

### 5.1. Perioperative Setting

The most consistent clinical evidence supporting immunonutrition originates from the perioperative context. Randomized trials and meta-analyses in high-risk surgical populations have demonstrated reductions in postoperative infectious complications and, in selected cohorts, shorter hospital stays when immunonutrition is initiated preoperatively and continued into the postoperative period [5,7]. These benefits appear most pronounced in major oncologic resections associated with substantial metabolic stress.

In head and neck oncology, extensive resections with reconstructive procedures generate comparable physiological demands. Surgical trauma induces cytokine release, transient insulin resistance, increased proteolysis, and elevated amino acid requirements for wound healing and immune function. Within this metabolic environment, provision of arginine- and omega-3-enriched formulations may help modulate inflammatory responses and support tissue repair. Accordingly, perioperative immunonutrition may be considered in high-risk surgical patients as part of structured nutritional management [5].

### 5.2. Conservative Oncologic Treatment (Radiotherapy/Chemoradiotherapy)

In contrast, the role of immunonutrition during definitive radiotherapy or chemoradiotherapy remains less clearly defined. Unlike acute surgical stress, chemoradiotherapy is characterized by cumulative mucosal injury, sustained inflammatory signaling, and progressive impairment of oral intake. Although immune-modulating substrates may theoretically attenuate inflammatory cascades, clinical data in this setting are heterogeneous and do not consistently demonstrate superiority over standard nutritional support [1].

These differences likely reflect distinct metabolic contexts. In the perioperative period, immunonutrition targets a relatively acute inflammatory surge and increased substrate demand for tissue repair. During chemoradiotherapy, however, catabolic processes may be driven by persistent mucosal toxicity and prolonged inflammatory burden, which are not necessarily reversed by substrate enrichment alone. This distinction underscores the importance of treatment-modality-specific nutritional strategies rather than uniform supplementation approaches.

### 5.3. Adjunctive Amino Acid–Targeted Strategies

Beyond classical immunonutrients, targeted provision of specific amino acids—particularly branched-chain amino acids (BCAA) and essential amino acids—has attracted interest as a potential strategy to attenuate surgery-induced muscle catabolism. BCAAs, especially leucine, play a central role in stimulating muscle protein synthesis through mTOR-mediated pathways and may help counterbalance proteolytic signaling in catabolic states.

In non-head-and-neck surgical populations, perioperative amino acid-enriched nutritional regimens have been associated with improved nitrogen balance and, in some reports, reduced postoperative complications. However, evidence remains heterogeneous and not uniformly supported by large randomized trials [7].

Observational data from anatomically related upper aerodigestive procedures further illustrate this concept. In a prospective study of patients undergoing tonsillectomy, supplementation with branched-chain amino acids during the early postoperative period was associated with improved preservation of fat-free mass compared with standard nutritional care [8]. Although tonsillectomy differs etiologically from oncologic resection, the model shares relevant features: localized pharyngeal injury, transient swallowing impairment, and inflammatory activation. These similarities provide a plausible mechanistic bridge to head and neck cancer surgery.

Nevertheless, robust head-and-neck-specific randomized evidence on perioperative BCAA supplementation remains limited. Current data should therefore be interpreted as hypothesis-generating rather than definitive. Future trials incorporating standardized body composition and functional endpoints are required to determine whether targeted amino acid supplementation offers clinically meaningful benefit beyond adequate protein provision.

### 5.4. Microbiome Considerations

Emerging research suggests interactions between dietary substrates, amino acid metabolism, and the gut microbiome. Experimental and clinical observations indicate that microbial composition and metabolite production may influence systemic inflammatory signaling and skeletal muscle metabolism [10,11,12]. These interactions provide a biologically plausible link between nutritional substrate delivery, immune modulation, and muscle homeostasis.

However, direct clinical evidence in head and neck cancer remains limited. While microbiome-oriented strategies represent a promising research direction, routine implementation in HNC supportive care is not yet supported by high-quality, disease-specific data. At present, microbiome considerations should be regarded as exploratory adjuncts rather than established therapeutic components.

## 6. Mucositis in the Context of Nutritional and Metabolic Vulnerability

Mucositis is among the most frequent and clinically consequential toxicities in patients undergoing radiotherapy or chemoradiotherapy for head and neck cancer. Although traditionally regarded as a local adverse effect of treatment, its broader nutritional and metabolic implications are often underestimated. In clinical reality, mucositis frequently contributes to reduced oral intake, inflammatory activation, and subsequent muscle depletion, rather than functioning solely as an isolated epithelial complication.

From a clinical perspective, mucositis often marks a turning point during treatment. Early in the therapeutic course, many patients maintain oral intake despite discomfort. As epithelial injury progresses, however, pain, ulceration, dysphagia, xerostomia, and taste disturbances frequently converge, resulting in a rapid and sometimes profound reduction in food consumption. Importantly, this decline in intake may precede measurable changes in body weight, particularly in individuals with preserved or elevated baseline BMI. Reliance on weight-based monitoring alone may therefore delay recognition of clinically meaningful nutritional deterioration [3].

The biological development of mucositis follows a multistep process characterized by epithelial damage, activation of nuclear factor-κB-mediated pathways, amplification of pro-inflammatory cytokines, ulceration, and subsequent healing [13]. Although these events originate locally, systemic inflammatory signaling may extend beyond the mucosa. Elevated cytokine activity has been associated with increased proteolysis and impaired muscle protein synthesis in catabolic states, thereby reinforcing skeletal muscle loss in vulnerable patients. Within this framework, mucositis contributes both directly—through reduced intake—and indirectly—through inflammatory activation—to progressive nutritional decline.

Clinical studies have shown that severe mucositis is associated with higher rates of treatment interruption, dose reduction, and hospitalization [14]. These disruptions may compromise oncologic efficacy and prolong recovery. From a nutritional standpoint, mucositis imposes a dual burden: functional limitation of alimentation and amplification of systemic catabolic signaling. Recognizing this dual impact is essential when designing supportive care strategies.

Preventive and supportive measures are therefore integral to multidisciplinary management. Optimized oral hygiene protocols, adequate analgesia, and early dietetic counseling are core components of care [15]. In addition, pre-treatment dental assessment and optimization represent an essential step before initiating radiotherapy. Elimination of active dental infection, management of periodontal disease, and stabilization or extraction of compromised teeth are recommended to reduce the risk of secondary infection and osteoradionecrosis during and after treatment [13,14]. Although dental sanitation does not prevent radiation-induced mucositis itself, structured oral care and dental optimization may reduce superimposed infectious complications and limit additional inflammatory burden, thereby indirectly supporting treatment tolerance and nutritional maintenance [14]. Integration of dental evaluation into pretreatment planning therefore carries clinically meaningful implications, particularly in patients at high risk for impaired oral intake.

When clinically significant mucositis develops, reliance on oral intake alone may become unrealistic. Timely escalation to enteral nutrition can prevent further nutritional deterioration and help maintain treatment continuity [1,5]. Importantly, nutritional support during this phase should address not only caloric adequacy but also sufficient protein provision, given the heightened risk of muscle loss in the presence of inflammatory stress [6].

The consequences of mucositis frequently extend beyond the acute treatment period. Persistent mucosal sensitivity, dysphagia, altered salivary function, and taste disturbances may continue after completion of therapy, limiting spontaneous recovery of oral intake. In this way, mucositis forms a bridge between acute toxicity and prolonged nutritional vulnerability. Ongoing monitoring of body composition and functional capacity during the transition to survivorship is therefore warranted to prevent unrecognized, sustained lean tissue depletion.

## 7. Post-Acute Recovery: Nutritional and Functional Restoration

The period following completion of oncologic therapy represents a physiologically distinct phase characterized by gradual transition from acute inflammatory stress toward tissue repair and functional adaptation. In head and neck cancer, recovery is frequently prolonged. Persistent dysphagia, altered taste perception, xerostomia, mucosal sensitivity, and reduced physical activity may continue beyond the active treatment phase, limiting restoration of adequate nutritional intake.

Importantly, muscle depletion acquired during radiotherapy, chemoradiotherapy, or surgery does not necessarily recover spontaneously once treatment has ended. Stabilization of body weight during follow-up may create the impression of nutritional recovery, yet body composition may remain unfavorable, with incomplete restoration of lean tissue. Given the established association between low skeletal muscle mass and adverse oncologic outcomes [3,4,9], post-treatment monitoring should extend beyond body weight alone.

In addition to persistent dysphagia and xerostomia, trismus and other structural or neuromuscular impairments of the head and neck region may further compromise oral intake and rehabilitation potential. Restricted mandibular mobility can substantially limit dietary diversity and caloric adequacy, thereby prolonging nutritional vulnerability beyond the acute treatment phase.

The post-acute phase should therefore be regarded as an active therapeutic window rather than a passive convalescent period. Re-establishment of sufficient protein intake and structured refeeding strategies may support recovery of muscle protein synthesis once inflammatory burden decreases. However, longitudinal data describing the trajectory of lean mass restoration in head and neck cancer populations remain limited, and high-quality prospective studies are needed.

Evidence from anatomically related surgical models illustrates how transient impairment of oral intake can influence body composition during recovery. In a prospective study of patients undergoing tonsillectomy, early postoperative supplementation with branched-chain amino acids was associated with improved preservation of fat-free mass compared with standard care [8]. Although etiologically distinct from oncologic surgery, the model shares clinically relevant features: localized pharyngeal injury, temporary swallowing impairment, and inflammatory activation. These parallels suggest that targeted metabolic support during recovery may warrant further investigation in head and neck oncology, particularly in patients with documented low skeletal muscle mass.

Nevertheless, robust randomized evidence specific to head and neck cancer survivors is currently lacking. Future trials should incorporate standardized body composition assessment together with functional measures such as muscle strength and performance to determine whether structured post-treatment nutritional strategies translate into durable improvements in physical resilience and quality of life.

Beyond nutritional composition alone, integration of dietetic follow-up with swallowing rehabilitation and gradual restoration of physical activity is likely essential for meaningful functional recovery. A coordinated multidisciplinary approach may reduce the risk of persistent muscle depletion and long-term functional impairment.

In summary, the post-acute phase represents a critical but often underrecognized component of supportive care in head and neck oncology. Continued monitoring of nutritional status, body composition, and functional capacity beyond active treatment is necessary to prevent sustained lean tissue depletion and to support long-term recovery.

A further challenge during survivorship is the fragmentation of care. Following discharge from specialized oncology units, patients may lose direct contact with their multidisciplinary inpatient teams. Access to coordinated nutritional counseling, swallowing therapy, and rehabilitation expertise in community or primary care settings is often limited. This transition gap may contribute to unrecognized persistence of muscle depletion and functional decline.

## 8. Clinical Implications and Future Directions

The evidence reviewed in this manuscript supports a reconceptualization of nutritional supportive care in head and neck cancer. Malnutrition, low skeletal muscle mass, mucositis, and inflammatory activation should not be approached as isolated complications but rather as interconnected components of a dynamic metabolic and functional continuum.

From a clinical perspective, early identification of nutritional vulnerability remains fundamental. Validated screening instruments such as the Patient-Generated Subjective Global Assessment and the Mini Nutritional Assessment provide practical tools for routine evaluation, while additional phenotypic characterization—where feasible—may refine detection of muscle depletion. Importantly, precise terminology is essential. The distinction between low skeletal muscle mass and formally defined sarcopenia, which requires functional impairment, should be maintained to ensure conceptual clarity and comparability across studies [4,9].

Timing consistently emerges as a determinant of outcome. Interventions initiated at diagnosis or at the beginning of treatment are more likely to preserve treatment intensity and functional capacity than strategies introduced reactively after substantial weight loss or severe toxicity has developed [1,5]. Within this framework, mucositis should be recognized not merely as a treatment-related adverse event but as a metabolic inflection point that may necessitate coordinated nutritional, analgesic, and oral care measures [15,16,17].

The perioperative period represents a setting in which immunonutrition has demonstrated measurable benefit in selected high-risk surgical populations [5,7]. However, extrapolation of these findings to all treatment modalities would be inappropriate. During chemoradiotherapy, where inflammatory stress is cumulative and sustained, the metabolic context differs substantially from acute surgical trauma. Nutritional strategies should therefore be adapted to treatment modality and clinical condition rather than applied uniformly.

The post-treatment phase warrants equal attention. Stabilization of body weight should not be equated with complete metabolic recovery. Persistent deficits in lean tissue and functional capacity may remain undetected without structured follow-up. Integration of body composition assessment, swallowing rehabilitation, and individualized dietary planning into survivorship pathways may reduce the risk of prolonged muscle depletion and long-term disability.

Future research should prioritize prospective studies incorporating standardized muscle mass measurement alongside functional endpoints such as strength, physical performance, and treatment tolerance. Trials evaluating adjunctive metabolic strategies—including amino acid-targeted supplementation or microbiome-informed approaches—should define clear phenotypic and functional outcomes to determine clinical relevance [8,10,11,12].

Ultimately, effective nutritional management in head and neck cancer requires alignment between pathophysiology, assessment methodology, and intervention timing. A body composition-oriented and inflammation-aware framework may offer a more precise basis for supportive care than weight-centered paradigms alone.

From a broader conceptual perspective, nutritional vulnerability in head and neck cancer may be viewed as a treatment-modality-dependent metabolic phenotype rather than a uniform condition. Surgical pathways are characterized predominantly by acute inflammatory surges and wound-healing demands, whereas chemoradiotherapy induces cumulative mucosal injury and sustained catabolic signaling. These differing biological contexts influence the trajectory of muscle depletion and recovery potential. Accordingly, structured nutritional assessment should not function merely as risk documentation but as a decision-guiding tool that informs modality-specific intervention. Recognizing this dynamic interplay between intake limitation, inflammatory activation, and muscle metabolism may help shift supportive care from reactive calorie replacement toward proactive metabolic stewardship across the entire treatment continuum.

## 9. Limitations

This review has limitations inherent to its narrative design. It does not follow a predefined systematic methodology, and therefore, study selection and interpretation may be subject to bias. The available literature in head and neck cancer is heterogeneous with respect to definitions of muscle depletion, assessment methods, intervention protocols, and outcome measures. In particular, objective functional parameters required for the formal diagnosis of sarcopenia are inconsistently reported across studies, limiting direct comparison.

Evidence regarding adjunctive metabolic strategies—including amino acid-targeted supplementation and microbiome-oriented approaches—remains preliminary in head and neck oncology. Much of the mechanistic rationale is extrapolated from related surgical or oncologic populations. While these analogies are physiologically plausible, robust head-and-neck-specific randomized data are still limited.

Finally, access to advanced body composition assessment may vary across institutions, potentially affecting feasibility of widespread implementation. Despite these constraints, the integration of available clinical and mechanistic evidence provides a structured framework for future investigation and clinical refinement.

## 10. Conclusions

Malnutrition, low skeletal muscle mass, and treatment-related mucosal toxicity remain central determinants of clinical trajectory in head and neck cancer. Their interaction reflects a complex interplay between impaired intake, inflammatory activation, and progressive muscle depletion rather than isolated nutritional imbalance.

Early identification of nutritional vulnerability, accurate phenotypic characterization, and timely intervention are essential for preserving treatment feasibility and functional capacity. Weight stability alone should not be interpreted as metabolic recovery, particularly in the presence of persistent inflammatory stress or impaired oral function.

Perioperative immunonutrition has demonstrated benefit in selected high-risk surgical settings, whereas its role during chemoradiotherapy requires careful contextualization. Adjunctive metabolic strategies, including amino acid-targeted supplementation and microbiome-informed approaches, remain areas of active investigation rather than established standard practice.

A supportive care framework that integrates structured screening, body composition awareness, modality-specific nutritional planning, and continued monitoring into the post-treatment phase may offer a more precise approach than calorie-centered paradigms alone. Future research should prioritize standardized phenotypic and functional endpoints to determine whether such strategies translate into durable improvements in resilience and long-term outcomes in head and neck oncology.

## Data Availability

No new data were created or analyzed in this study.
